# Duckweed (*Lemnaceae*) as a Functional Protein Ingredient in Koi Carp Diets: Species‐Dependent Effects on Growth, Pigmentation, Antioxidant Status, and Gut Health

**DOI:** 10.1155/anu/3873558

**Published:** 2026-06-07

**Authors:** Yingjie Song, Shasha Luo, Shuang Li, Xinyan Yang, Ruimin He, Zhangli Hu, Xuewei Yang, Yinglin Lu

**Affiliations:** ^1^ Institute of Nanfan and Seed Industry, Guangdong Academy of Sciences, Guangzhou, 510220, China, gdas.gd.cn; ^2^ Guangdong Provincial Key Laboratory of Research on Functions and Preventive Medicine of Medicinal and Edible Resources in Eastern Guangdong, Hanshan Normal University, Chaozhou, 521000, China, hstc.edu.cn; ^3^ College of Life Sciences and Oceanography, Shenzhen University, Shenzhen, 518060, China, szu.edu.cn

**Keywords:** antioxidant activity, circular bioresource, duckweed proteins, functional ingredients, gut microbiota

## Abstract

Duckweed (*Lemnaceae*) has emerged as a promising aquatic plant protein for aquafeeds, yet its functional impacts on ornamental fish remain insufficiently characterized. This study evaluated five duckweed species, *Lemna minor* (LM), *Wolffia globosa* (WO), *Landoltia punctata* (LP), *Spirodela polyrhiza* (SP), and rootless *L. punctata* (LR), as partial fishmeal replacers in koi carp (*Cyprinus carpio*). Phase I determined optimal inclusion levels based on growth performance using isonitrogenous and isolipidic diets (0–40% weight‐for‐weight [w/w]). Phase II further assessed selected inclusions (LM_20_, WO_20_, LP_30_, SP_10_, and LR_30_) for pigmentation, antioxidant status, intestinal immunity, and gut microbiota. Moderate replacement with LM and WO (20%) significantly improved weight gain (WG), feed conversion ratio (FCR), protein digestibility, and essential amino acid retention compared with the control. These treatments also enhanced skin redness and yellowness, accompanied by higher muscle astaxanthin, zeaxanthin, and lutein deposition. Antioxidant defenses (superoxide dismutase [SOD] and catalase [CAT]) were elevated and lipid peroxidation (malondialdehyde [MDA]) reduced, while intestinal lysozyme and phenoloxidase (PO) activities increased. Proinflammatory cytokines (tumor necrosis factor‐α [TNF‐α] and interleukin‐1β [IL‐1β]) were downregulated, whereas IL‐10 and tight‐junction genes (claudin, occludin, and ZO‐1) were upregulated, indicating improved mucosal immunity and barrier function. Gut microbiota profiling revealed enriched *Lactobacillus* and *Bacillus* and higher α‐diversity in LM_20_ and WO_20_, whereas SP (SP_10_) showed limited benefits. These findings demonstrate that specific duckweed species, particularly LM and WO, act as functional ingredients beyond protein supply, improving pigmentation, antioxidant capacity, mucosal immunity, and beneficial gut microbial profiles. These results identify LM and WO as nutritionally feasible and functionally active protein ingredients for koi carp, supporting their application in sustainable ornamental aquafeeds.

## 1. Introduction

Global aquaculture production continues to expand rapidly to satisfy the increasing human demand for high‐quality animal protein, consequently escalating the requirement for high‐protein ingredients in aquafeeds, particularly fishmeal [1]. Traditionally, fishmeal has been extensively utilized in aquaculture diets owing to its excellent amino acid profile, high digestibility, and palatability, supporting optimal growth and health performance of various aquatic species [2]. However, reliance on fishmeal is increasingly problematic due to its limited supply, rising prices, and significant environmental concerns, including overfishing, declining wild fish stocks, and habitat destruction associated with wild fish harvesting [3].

Plant‐derived proteins, such as soybean meal, rapeseed meal, cottonseed meal, and sunflower seed meal, have been extensively researched as sustainable alternatives for fishmeal replacement. However, the application of these traditional plant‐based ingredients is often constrained by factors including imbalanced amino acid profiles, low digestibility, poor palatability, and the presence of antinutritional factors (ANFs), such as trypsin inhibitors, phytic acid, tannins, and glucosinolates, which adversely impact fish health and growth performance [4–6]. Thus, it remains essential to identify novel plant‐based ingredients with superior nutritional profiles, minimal ANFs, and environmental sustainability to enhance the effectiveness of aquafeed formulations.

Recently, duckweed species belonging to the *Lemnaceae* family, such as *Lemna minor (LM)*, *Wolffia globosa (WO)*, *Landoltia punctata (LP)*, and *Spirodela polyrhiza (SP)*, have attracted considerable attention as promising alternatives to conventional protein sources due to their high biomass yield, rapid growth rate, favorable amino acid profile, and minimal cultivation footprint [7, 8]. Several recent studies have reported duckweed crude protein content ranging from ~25% to over 45% on a dry matter basis, comparable or superior to conventional plant proteins [9, 10]. Duckweed proteins generally exhibit relatively balanced essential amino acid profiles compared with many conventional plant ingredients, although the abundance of individual amino acids may vary among species, cultivation conditions, and processing methods. Therefore, in practical feed formulation, crystalline amino acids may still be required to achieve an appropriate amino acid balance when duckweed is incorporated at different inclusion levels [11, 12].

Moreover, duckweed cultivation provides additional environmental benefits, including effective removal of nitrogen and phosphorus from wastewater, thereby potentially reducing the ecological footprint of aquaculture operations and improving water quality [13, 14]. Despite these advantages, the presence of ANFs in duckweed, such as phytic acid, oxalic acid, and tannins, may negatively affect nutrient digestibility, intestinal health, and overall fish performance if dietary inclusion levels are not adequately optimized [15]. Thus, further research is critical to systematically evaluate optimal inclusion rates that maximize duckweed’s beneficial effects while minimizing potential negative impacts.

Koi carp (*Cyprinus carpio*), an ornamental variant of common carp, is globally popular and economically valuable due to its esthetic characteristics, including vibrant and diverse pigmentation patterns [16, 17]. Maintaining and enhancing the growth, health, immune function, and especially coloration of koi carp are essential considerations in ornamental aquaculture. Although recent studies have explored the potential of various plant‐based proteins and dietary additives in koi diets to promote growth and pigmentation [18], comprehensive evaluations of duckweed as a sustainable protein replacement, particularly focusing on its effects on intestinal health, immune function, and pigmentation, are limited.

Previous research indicated that dietary carotenoids and antioxidants could significantly improve pigmentation and health status in ornamental fish [16, 18]. Duckweed species naturally contain substantial amounts of carotenoids such as lutein and zeaxanthin, suggesting their potential to enhance fish coloration [7]. However, systematic studies assessing these effects in koi carp are scarce, particularly those involving a comparative evaluation among multiple duckweed species.

Therefore, the present study was designed to comprehensively assess the potential of dietary fishmeal replacement with various duckweed species (LM, WO, LP, SP, and rootless *L. punctata (LR)*) in koi carp diets. The objectives of this research were to evaluate the impacts of duckweed‐based diets on growth performance, nutrient digestibility, antioxidant enzyme activity, intestinal immune response, gut barrier integrity, and skin pigmentation. Furthermore, the study aimed to elucidate possible underlying nutritional and physiological mechanisms influencing these effects, ultimately providing scientific evidence supporting the practical use of duckweed in aquafeeds. We hypothesized that specific duckweed species could effectively replace a substantial proportion of fishmeal without adverse effects, potentially improving koi carp’s antioxidant capacity, immune function, intestinal barrier integrity, and pigmentation. The outcomes from this research are expected to contribute valuable insights into the sustainable utilization of duckweed resources in aquafeed formulation, advancing environmentally friendly aquaculture practices. Unlike previous studies focusing on single duckweed species or growth performance alone, this study applies a two‐phase nutritional screening framework to identify species‐specific and inclusion‐dependent functional effects of duckweed in koi carp diets.

## 2. Materials and Methods

### 2.1. Experimental Fish and Rearing Conditions

Healthy koi carp (*C. carpio*) juveniles, initially weighing 12.5 ± 0.2 g, were obtained from a commercial hatchery. Prior to the experiment, fish were acclimated in laboratory conditions for 2 weeks and fed a commercial koi diet (protein content ≥ 40% and fat ≥ 6%). After acclimation, 540 fish were randomly allocated to 18 fiberglass tanks (300 L), each tank containing 30 fish. Each diet treatment was replicated three times. The experiment lasted for 8 weeks, with fish fed three times daily (8:00, 12:00, and 17:00) to apparent satiation. Water temperature was maintained at 25 ± 1°C, dissolved oxygen ≥ 6.0 mg/L, pH 7.0–7.5, ammonia nitrogen ≤ 0.05 mg/L, and photoperiod set at 12‐h light/dark cycle.

### 2.2. Experimental Design and Dietary Treatments

The five duckweed meals used in this study were obtained from the duckweed germplasm collection maintained at the Institute of Nanfan and Seed Industry, Guangdong Academy of Sciences, Guangzhou, China. All biomasses were produced under greenhouse tank culture conditions using freshwater supplemented with Hoagland nutrient solution and were harvested at the vegetative growth stage. Before feed preparation, the duckweed biomass was rinsed to remove adhering particles, dried at 60°C to constant weight, ground to pass through a 60‐mesh sieve, and stored at −20°C until use. The internal line IDs used in this study were as follows: LM (LM‐GAS‐01), WO (WO‐GAS‐01), LP (LP‐GAS‐01), SP (SP‐GAS‐01), and LR (LR‐GAS‐01). The LR used in this study was maintained as a separate line in the same germplasm collection and propagated independently from the rooted LP line. Because duckweed composition is strongly influenced by cultivation environment, the proximate composition and ANF contents of each duckweed meal were determined prior to formulation and are reported in Table S3.

#### 2.2.1. Phase I—Evaluation of Optimal Replacement Levels

In Phase I, five duckweed species, namely, LM, WO, LP, SP, and LR, were evaluated individually at five dietary inclusion levels (0%, 10%, 20%, 30%, and 40%, weight‐for‐weight [w/w]). In this experimental design, duckweed inclusion levels were defined on a w/w basis in the diet, and the added fishmeal fraction was reduced proportionally on the same basis. However, this does not imply that duckweed and fishmeal were assumed to have identical crude protein or crude lipid contents. After setting the intended duckweed inclusion level, the remaining dietary ingredients were adjusted to produce practical diets that were approximately isonitrogenous (~40% crude protein) and isolipidic (~6% crude lipid). Wheat flour and crystalline amino acids (L‐lysine and DL‐methionine) were used as needed to maintain overall nutrient balance, to standardize essential amino acid supply among diets, and to minimize confounding effects arising from amino acid imbalance. This formulation strategy was adopted to allow a clearer comparison of duckweed species and inclusion levels within practical, nutritionally balanced aquafeed formulations, rather than to specifically test the independent effects of amino acid deficiency. Therefore, the Phase I diets should be interpreted as weight‐based fishmeal reduction within nutritionally rebalanced formulations, rather than as direct protein‐equivalent substitution of fishmeal by duckweed. The ingredient composition and proximate analysis of the 25 Phase I diets are presented in Table S1, and proximate composition was verified according to AOAC methods.

Each diet was assigned to three replicates (*n* = 3), and 30 fish were randomly stocked into each of 75 fiberglass tanks (300 L). Fish were fed the respective diets to apparent satiation three times daily (08:00, 12 :00, and 17:00) for 8 weeks.

Growth performance was evaluated using the following indicators: weight gain (WG), specific growth rate (SGR), feed conversion ratio (FCR), and survival rate (SR). The optimal replacement level for each duckweed species was determined based on the highest growth performance and lowest FCR without compromising survival and used in the second phase.

#### 2.2.2. Phase II—Comparative Evaluation of Optimal Inclusion Levels

Based on Phase I results, the following inclusion levels were selected for further in‐depth study:

• LM_20_: LM at 20% inclusion (w/w)
• WO_20_: WO at 20% inclusion (w/w)
• LP_30_: LP at 30% inclusion (w/w)
• SP_10_: SP at 10% inclusion (w/w)
• LR_30_: LR at 30% inclusion (w/w)
• Cont._0_: control (0% replacement, fishmeal only)

These inclusion levels correspond to proportional reductions of the added fishmeal fraction on a weight basis. After this replacement step, the remaining ingredients were adjusted to obtain diets that were approximately isonitrogenous and isolipidic.

For Phase II, six practical diets corresponding to the Phase II treatments (control, LM_20_, WO_20_, LP_30_, SP_10_, and LR_30_) were formulated. Duckweed inclusion was defined on a weight basis, whereas overall dietary protein and lipid levels were rebalanced during formulation to achieve ~40% crude protein and 6% crude lipid. Diet ingredient compositions are provided in Table S2. Essential amino acid balance was maintained across treatments using crystalline L‐lysine and DL‐methionine as needed. All ingredients were ground (<0.25 mm), mixed, pelleted (2 mm), air‐dried (<45°C), and stored at –20°C prior to feeding. Proximate composition of finished feeds confirmed targets of ~40% crude protein and ~6% crude lipid. The proximate and amino acid composition of each duckweed meal used for formulation is reported in Table S1, and the calculated essential amino acid profiles of Phase II diets are summarized in Table S2.

Each of the six diets was administered to koi carp for 8 weeks (*n* = 3 tanks per treatment, 30 fish per tank). Growth performance, feed utilization, whole‐body composition, amino acid profiles, pigmentation, serum biochemical markers, antioxidant enzyme activities, intestinal immunity, and tight junction gene expression were analyzed. The Phase II design was intended to compare the physiological and functional responses of koi carp to the optimal inclusion level identified for each duckweed type under nutritionally comparable diet conditions.

### 2.3. Sampling and Sample Preparation

At the end of the Phase II feeding trial, fish were fasted for 24 h to ensure gut clearance. All surviving fish were counted and group‐weighed to calculate growth and feed utilization metrics. Six fish were randomly selected from each tank and euthanized using MS‐222 (100 mg/L). Selection was based on random netting to avoid bias, and body weight distributions of sampled fish did not differ significantly from the tank averages (*p* > 0.05). Thus, the subsamples were representative of each group. Blood samples were collected from the caudal vein using 1 mL syringes and transferred to nonheparinized tubes. Serum was separated by centrifugation at 3500 rpm for 10 min at 4°C and stored at –80°C for biochemical and antioxidant analyses.

From each selected fish, dorsal muscle and skin were excised for amino acid and pigment analysis, snap‐frozen in liquid nitrogen, and stored at –80°C. The mid‐intestine was also collected aseptically; one portion was stored in RNAlater (Invitrogen) for RNA extraction and gene expression analysis, while the remaining intestinal tissue was frozen for enzyme activity assays.

### 2.4. Growth Performance and Nutrient Utilization

Growth performance parameters were calculated using standard equations. As below:

• WG (%) = 100 × (Final body weight − Initial body weight)/Initial body weight;
• Specific growth rate (SGR, %/day) = 100 × [ln(Final body weight) − ln(Initial body weight)]/Feeding days;
• Feed conversion ratio (FCR) = Total dry feed intake/Total wet WG;
• Survival rate (SR, %) = 100 × Final fish number/Initial fish number.

### 2.5. Nutritional and Amino Acid Analyses

Proximate composition of duckweed powders and formulated diets was determined following AOAC (2016) methods. The proximate and amino acid composition of each duckweed species used as feed ingredients are presented in Table S3, providing a nutritional basis for interpreting the effects of inclusion levels in Phases I and II. Moisture content was analyzed by oven‐drying at 105°C, crude protein by Kjeldahl nitrogen (N × 6.25), crude lipid by Soxhlet extraction using petroleum ether, and ash content by combustion in a muffle furnace at 550°C for 6 h. Muscle amino acid composition was analyzed by hydrolyzing muscle samples in 6 M HCl at 110°C for 24 h, followed by derivatization with phenyl isothiocyanate (PITC) and quantification via high‐performance liquid chromatography (HPLC) [19]. Tryptophan was determined separately after alkaline hydrolysis.

### 2.6. Skin Color and Pigment Deposition

Skin color parameters including lightness (*L* 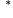), redness (*a* 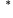), and yellowness (*b* 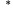) were measured using a calibrated Minolta CR‐400 chroma meter following previously described procedures for koi color evaluation, with minor modifications [18]. For pigment analysis, carotenoids (astaxanthin, zeaxanthin, and lutein) were extracted from homogenized muscle tissue using acetone/ethanol solvent and quantified by HPLC following previously described approaches for ornamental fish pigmentation studies, with minor modifications [18]. Carotenoids were identified and quantified using authentic standards and expressed as mg/kg dry weight.

### 2.7. Serum Biochemical Parameters

Serum total protein (TP), aspartate aminotransferase (AST), and alanine aminotransferase (ALT) levels were determined using commercial diagnostic kits (Mindray, China) and measured with an automatic biochemistry analyzer (Mindray BS‐420). Antioxidant capacity was assessed by measuring superoxide dismutase (SOD) and catalase (CAT) activities and malondialdehyde (MDA) concentration using colorimetric assay kits (Nanjing Jiancheng Bioengineering Institute, China) according to manufacturer instructions. Serum biochemical and antioxidant parameters were determined according to the manufacturers’ instructions and in line with previously reported applications in fish physiological assessment [20].

### 2.8. Antioxidant and Immune Parameters

Intestinal lysozyme activity was determined using a turbidimetric method based on the lysis rate of *Micrococcus lysodeikticus*. Phenoloxidase (PO) activity was assessed using L‐DOPA substrate and measuring absorbance change at 490 nm [21]. Total RNA from mid‐intestinal tissues was extracted using TRIzol reagent (Invitrogen, USA), and RNA purity was confirmed by the A260/A280 ratio. cDNA was synthesized from 1 µg RNA using a PrimeScript RT kit (Takara, Japan). Quantitative real‐time PCR (qPCR) was performed using SYBR Green Master Mix (Bio‐Rad, USA) on a CFX96 real‐time PCR system.

Target genes included the proinflammatory cytokines tumor necrosis factor‐α (TNF‐α) and interleukin‐1β (IL‐1β), anti‐inflammatory cytokine interleukin‐10 (IL‐10), and tight junction proteins claudin, occludin, and ZO‐1. Two reference genes (β‐actin and EF1α) were validated for stability using geNorm (*M* < 0.5). Amplification efficiencies ranged 92%–106%, *R* ^2^ > 0.99. Relative expression was normalized to the geometric mean of both reference genes (2^−ΔΔCt^) [22]. qPCR efficiencies (E %) and standard curves for all target and reference genes are provided in Figure S1 and Table S4.

### 2.9. ANF Determination

Phytic acid content was measured by the Wade reagent method based on the formation of a pink complex with ferric chloride [23]. Oxalate concentration was determined by acid extraction followed by titration with potassium permanganate [24]. Tannin levels were assessed using the vanillin‐HCl method [25]. All chemical determinations were performed in triplicate.

### 2.10. Gut Microbiota Analysis

At the end of the feeding trial, mid‐intestinal digesta from three fish per tank (*n* = 3) were aseptically collected and pooled into one composite sample per replicate for microbial DNA analysis. All samples were immediately frozen in liquid nitrogen and stored at –80°C until processing. Total microbial genomic DNA was extracted using the E.Z.N.A. Soil DNA Kit (Omega Bio‐tek, Norcross, GA, USA) following the manufacturer’s instructions, optimized for intestinal content. DNA integrity and purity were assessed by agarose gel electrophoresis and spectrophotometry (NanoDrop 2000, Thermo Scientific, USA).

The V3–V4 hypervariable regions of the bacterial 16S rRNA gene were amplified using universal primers 341F (5^′^‐CCTAYGGGRBGCASCAG‐3^′^) and 806R (5^′^‐GGACTACNNGGGTATCTAAT‐3^′^) with Illumina adapter overhangs. PCR amplification was performed in triplicate using a high‐fidelity polymerase (Phusion, New England Biolabs, USA), and the amplicons were purified using AMPure XP beads (Beckman Coulter, USA). Library construction and paired‐end sequencing (2 × 250 bp) were conducted on the Illumina MiSeq platform (Illumina, San Diego, CA, USA).

Raw reads were processed using the QIIME2 (version 2022.2) pipeline [26]. After quality filtering, denoising, merging, and chimera removal, representative amplicon sequence variants (ASVs) were assigned taxonomic identities using the SILVA 138 reference database [27]. Alpha‐diversity indices (Chao1, Shannon, and Simpson) and beta diversity (Bray–Curtis dissimilarity and principal coordinate analysis [PCoA]) were calculated to assess microbial richness and community structure. Differentially abundant taxa were identified using linear discriminant analysis effect size (LEfSe), with a logarithmic LDA score threshold of 2.0 and *p* < 0.05 as significance cutoff [28].

To further understand the functional implications of gut microbial shifts, Phylogenetic Investigation of Communities by Reconstruction of Unobserved States (PICRUSt2) was employed to predict functional profiles of microbial communities, focusing on pathways related to immune modulation, nutrient metabolism, and oxidative stress response [29].

### 2.11. Statistical Analysis

All data were expressed as mean ± standard error (SE). Statistical analyses were conducted using SPSS version 26.0 (IBM, USA). Differences among treatment groups were evaluated by one‐way analysis of variance (ANOVA). Statistical significance was accepted at *p* < 0.05. Pearson correlation analysis was also performed to explore associations between ANF contents and intestinal immune or antioxidant parameters. For Phase I (5 × 5 factorial design: species × inclusion level), data were analyzed using two‐way ANOVA to test the main effects of duckweed species and inclusion level, as well as their interaction. When significant effects were detected (*p* < 0.05), polynomial contrasts (linear and quadratic) were used to examine dose–response trends across inclusion levels. Tukey’s HSD test was employed for pairwise comparisons. Phase II data were analyzed separately by one‐way ANOVA followed by Tukey’s HSD.

## 3. Results

### 3.1. Growth Performance and Feed Utilization

**Figure 1:**
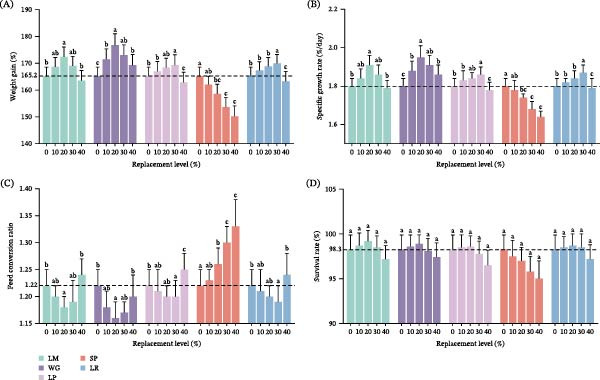
Growth performance of koi carp fed diets with graded duckweed inclusion (w/w) replacing fishmeal proportionally on a weight basis. (A) Weight gain (WG, %). (B) Specific growth rate (SGR, %/day). (C) Feed conversion ratio (FCR). (D) Survival rate (%). The dashed line represents the mean value of the control group. Values are presented as mean ± SD (*n* = 3). Different superscript letters indicate significant differences among treatments (*p* < 0.05).

Growth performance of koi carp fed diets with graded duckweed inclusion (w/w), which reduced fishmeal on a weight basis rather than protein equivalence, is shown in Figure 1. SRs remained consistently high across all treatments (>95%) with no significant differences among groups (*p* > 0.05; Figure 1D), indicating that duckweed inclusion did not compromise fish survival. WG varied across the 25 dietary treatments (Figure 1A; different superscript letters indicate significant differences among treatments, *p* < 0.05). WG recorded in the control group (165.2%) was used as a reference for comparison. The highest WG occurred with WO at 20% and 30% replacement (WO_20_, 176.8 ± 4.2%; WO_30_, 173.1 ± 3.8%), followed closely by LM at 20% and 30% (LM_20_, 172.4 ± 3.7%; LM_30_, 169.0 ± 3.6%). For LP, WG at LP_20_ and LP_30_ was 168.4 ± 3.5% and 169.3 ± 3.9%, respectively, remaining close to the control reference. LP_20_ was lower than LM_20_, whereas LP_30_ was comparable to LM_30_, indicating an overall intermediate growth response. LR maintained WG slightly above the reference (LR_20_,168.9 ± 3.7%; LR_30_, 170.0 ± 3.3%). SP showed a clear dose‐dependent decline: SP_10_ with 162.1 ± 3.1%, SP_30_ with 158.6 ± 3.6%, and SP_40_ with 150.2 ± 3.9%, the latter two being the lowest among all treatments.

SGR followed the same pattern (Figure 1B; reference line 1.80%/day). WO_20–30_ with 1.91–1.95%/day and LM_20–30_ with 1.86–1.92%/day were the top performers and were equal to or above the control. LP_20–30_ with 1.84–1.86%/day formed an intermediate tier. LR_20–40_ with 1.82–1.87%/day remained comparable to the control. In contrast, SP_30–40_ with 1.64–1.68%/day were clearly lower than most other treatments.

FCR largely mirrored growth responses (Figure 1C; reference line 1.22). LM_20–30_ and WO_20–30_ yielded the most favorable values (1.16–1.19), LP_20–30_ and LR_20–30_ were close to the reference (1.19–0.20), whereas SP_30–40_ exhibited markedly higher FCR (1.30–1.33), indicating poor feed efficiency at high SP inclusion.

Taken together, the treatment means shown in Figure 1 indicate that LM_20–30_ and WO_20–30_ produced the most favorable growth responses, with higher WG and SGR together with lower FCR. LP_20–30_ and LR_20–30_ remained close to the control, whereas SP_30–40_ showed reduced WG and SGR and increased FCR. On this basis, five diets (LM_20_, WO_20_, LP_30_, SP_10_, and LR_30_) were selected for subsequent evaluation.

### 3.2. Nutrient Digestibility, Whole‐Body Composition, and Muscle Amino Acids

**Figure 2:**
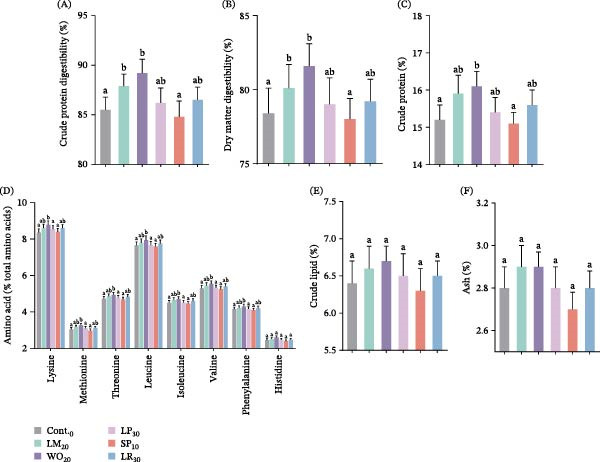
Nutrient digestibility, whole‐body proximate composition, and muscle amino acid profile of koi carp fed diets with selected duckweed inclusions. (A) Apparent crude protein digestibility (CPD, %). (B) Dry matter digestibility (DMD, %). (C) Whole‐body crude protein (%). (D) Muscle amino acid composition (percentage of total amino acids). (E) Whole‐body crude lipid (%). (F) Whole‐body ash (%). Values are presented as mean ± SD (*n* = 3). Different superscript letters indicate significant differences among treatments (*p* < 0.05).

Apparent digestibility coefficients of crude protein digestibility (CPD) and dry matter digestibility (DMD) are presented in Figure 2A, B. CPD in the control diet was ~85%, whereas significantly higher values were observed in LM_20_ (87.9%), WO_20_ (89.2%), and LP_30_ (86.2%) (*p* < 0.05). In contrast, SP_10_ (84.8%) remained comparable to the control, and LR_30_ (86.5%) showed intermediate values. A similar trend was recorded for DMD, with the highest values in WO_20_ (81.6%), followed by LM_20_ and LP_30_ (80.1% and 79.0%), while control and SP_10_ were the lowest (78%) (*p* < 0.05). These results indicate that replacing fishmeal with WO_20_, LM_20_, or LP_30_ improved nutrient digestibility, whereas SP_10_ did not confer any advantage.

Whole‐body proximate composition is shown in Figure 2C, E, F. Crude protein content ranged from 15.1% to 16.1%, with WO_20_ (16.1%) significantly higher than the control (15.2%) and SP_10_ (15.1%) (*p* < 0.05). LM_20_, LP_30_, and LR_30_ maintained intermediate values (15.4%–15.9%) with no significant differences among them. Crude lipid (6.3%–6.7%) and ash (2.7%–2.9%) contents did not differ significantly among treatments (*p* > 0.05). These findings suggest that moderate duckweed inclusion, particularly WO_20_, enhanced protein deposition without affecting lipid or mineral balance.

The amino acid composition of muscle is presented in Figure 2D. Lysine and methionine proportions were significantly higher in WO_20_ compared with the control and SP_10_ (*p* < 0.05). Leucine and isoleucine also tended to be elevated in WO_20_ and LP_30_ relative to SP_10_, whereas threonine, valine, phenylalanine, and histidine showed no marked variation across groups. Thus, WO_20_ consistently improved essential amino acid retention, while LM_20_ and LP_30_ maintained balanced amino acid profiles comparable to the control. Taken together, these results demonstrate that moderate replacement levels of LM_20_, WO_20_, and LP_30_ not only improved nutrient digestibility but also supported favorable body protein content and amino acid balance, whereas SP_10_ and LR_30_ showed limited benefits. These findings further validate the selection of LM_20_, WO_20_, LP_30_, SP_10_, and LR_30_ for subsequent evaluation of physiological and health‐related responses.

### 3.3. Effects of Duckweed Inclusion on Skin Coloration and Carotenoid Deposition

**Figure 3:**
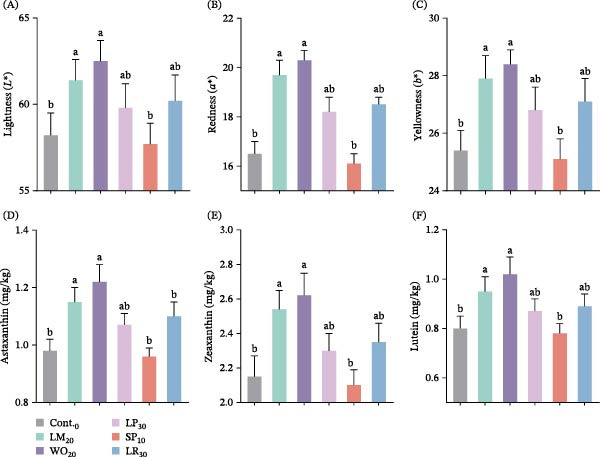
Skin coloration and carotenoid deposition in koi carp fed diets with selected duckweed inclusions. (A) Lightness (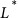). (B) Redness (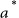). (C) Yellowness (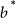). (D) Muscle astaxanthin (mg/kg). (E) Muscle zeaxanthin (mg/kg). (F) Muscle lutein (mg/kg). Values are presented as mean ± SD (*n* = 3). Different superscript letters indicate significant differences among treatments (*p* < 0.05).

Skin color parameters (Figure 3A–C) responded strongly to duckweed inclusion. Lightness (*L* 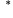) increased with LM_20_ (61.4) and WO_20_ (62.5) relative to the control (58.2; *p* < 0.05), while SP_10_ (57.7) remained low; LP_30_ (59.8) and LR_30_ (60.2) were intermediate. Redness (*a* 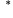) was highest in WO_20_ (20.3) and LM_20_ (19.7) and lowest in SP_10_ (16.1) and the control (16.5; *p* < 0.05), with LP_30_/LR_30_ (18.2 and 18.5) in between. Yellowness (*b* 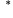) followed the same pattern: WO_20_ (28.4) and LM_20_ (27.9) exceeded the control (25.4) and SP_10_ (25.1) (*p* < 0.05), whereas LP_30_/LR_30_ (26.8 and 27.1) were intermediate. Thus, LM_20_ and WO_20_ consistently enhanced both red (*a* 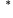) and yellow (*b* 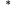) components of skin coloration, whereas SP_10_ produced the palest and least chromatic fish.

Muscle carotenoid contents (Figure 3D–F) corroborated the colorimetric data. Astaxanthin was greatest in WO_20_ (1.22 mg/kg) and LM_20_ (1.15 mg/kg), intermediate in LP_30_/LR_30_ (1.07 and 1.1 mg/kg), and lowest in SP_10_ (0.96 mg/kg) (*p* < 0.05). Zeaxanthin was likewise highest in WO_20_ (2.62 mg/kg) and LM_20_ (2.54 mg/kg), moderate in LP_30_/LR_30_ (2.3 and 2.35 mg/kg) and the control (2.15 mg/kg), and lowest in SP_10_ (2.1 mg/kg) (*p* < 0.05). Lutein showed the same ranking, WO_20_ (1.02 mg/kg) > LM_20_ (0.95 mg/kg) > LP_30_/LR_30_ (0.87 and 0.89 mg/kg) > control (0.8 mg/kg) > SP_10_ (0.78 mg/kg), with significant advantages for WO_20_ and LM_20_ (*p* < 0.05).

In summary, moderate replacement with WO_20_ and LM_20_ yielded the most vivid coloration (higher *a* 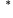 and *b* 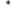) accompanied by greater deposition of astaxanthin, zeaxanthin, and lutein; LP_30_ and LR_30_ produced intermediate responses, and SP_10_ consistently gave the lowest pigmentation and carotenoid levels.

### 3.4. Serum Biochemistry and Antioxidant Status in Koi Carp Fed Selected Duckweed Diets

**Figure 4:**
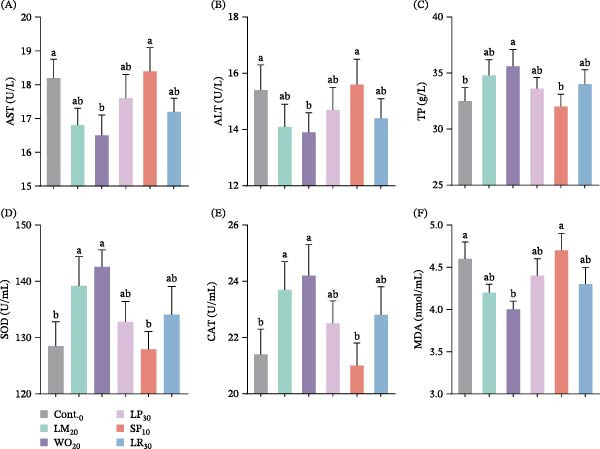
Serum biochemical indices and antioxidant status of koi carp fed diets with selected duckweed inclusions. Enzymatic activity of (A) aspartate aminotransferase (AST, U/L), (B) alanine aminotransferase (ALT, U/L), (C) total protein (TP, g/L), (D) superoxide dismutase (SOD, U/mL), (E) catalase (CAT, U/mL), and (F) malondialdehyde (MDA, mmol/mL). Values are presented as mean ± SD (*n* = 3). Different superscript letters indicate significant differences among treatments (*p* < 0.05).

Serum indicators of hepatic function and protein status are shown in Figure 4A–C. AST activity was lowest in WO_20_ (16.5 U/L), significantly below the control (18.2 U/L) and SP_10_ (18.4 U/L), with LM_20_, LP_30_, and LR_30_ at intermediate levels (16.8 U/L, 17.6 U/L, and 17.2 U/L) (*p* < 0.05). ALT displayed the same pattern: WO_20_ (13.9 U/L) < control (15.4 U/L) < SP_10_ (15.6 U/L), with LM_20_/LP_30_/LR_30_ intermediate (14.1 U/L, 14.7 U/L, and 14.4 U/L) (*p* < 0.05). TP was highest in WO_20_ (35.6 g/L), followed by LM_20_/LP_30_/LR_30_ (34.0–34.8 g/L), and lowest in the control and SP_10_ (32.0 g/L) (*p* < 0.05). Overall, these data indicate that WO_20_ and to a lesser extent LM_20_, LP_30_, and LR_30_ improved serum protein status and reduced hepatic enzyme activities compared with the control and SP_10_.

Antioxidant responses (Figure 4D–F) showed clear species effects. SOD activity increased in LM_20_ (139.2 U/L) and WO_20_ (14.2 U/L) relative to the control (128.5 U/L) and SP_10_ (127.9 U/L), with LP_30_/LR_30_ intermediate (132.8 U/L and 134.1 U/L). CAT was likewise higher in LM_20_ (23.7 U/L) and WO_20_ (24.2 U/L) than in the control (21.4 U/L) and SP_10_ (21.0 U/L); LP_30_/LR_30_ were intermediate (22.5 U/L and 22.8 U/L). Conversely, MDA concentrations were lowest in WO_20_ (4.0 mmol/mL), intermediate in LM_20_/LR_30_ (4.2 mmol/mL and 4.3 mmol/mL) and LP_30_ (4.4 mmol/mL), and highest in the control and SP_10_ (4.7 mmol/mL) (*p* < 0.05). These results suggest that WO_20_ and LM_20_ enhanced antioxidant defenses and reduced oxidative stress, while LP_30_ and LR_30_ produced intermediate effects, and SP_10_ did not differ from the control. Taken together, these findings demonstrate that moderate replacement with WO_20_ and LM_20_ improved serum biochemical indicators and antioxidant status in koi carp, whereas LP_30_ and LR_30_ were largely neutral, and SP_10_ offered no clear benefit.

### 3.5. Intestinal Innate Immunity and Barrier‐Related Gene Expression in Koi Carp Fed Selected Duckweed Diets

**Figure 5:**
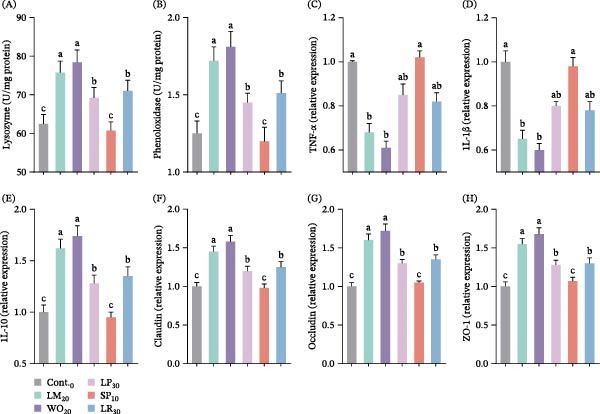
Intestinal immune enzyme activities and gene expression in koi carp fed diets with selected duckweed inclusions. (A) Lysozyme (U/mg protein). (B) Phenoloxidase (U/mg protein). (C) TNF‐α expression (relative to β‐actin). (D) IL‐1β expression (relative to β‐actin). (E) IL‐10 expression (relative to β‐actin). (F) Claudin expression. (G) Occludin expression. (H) ZO‐1 expression. Values are presented as mean ± SD (*n* = 3). Different superscript letters indicate significant differences among treatments (*p* < 0.05).

Activities of intestinal immune enzymes are shown in Figure 5A, B. Lysozyme activity was significantly higher in LM_20_ and WO_20_ (75.8 and 78.4 U/mg protein) compared with the control (62.5 U/mg protein) and SP_10_ (60.7 U/mg protein) (*p* < 0.05). LP_30_ and LR_30_ (69.2 and 71.0 U/mg protein) showed intermediate values. PO activity followed a similar trend, with LM_20_ and WO_20_ (1.72 and 1.81 U/mg protein) higher than the control and SP_10_ (1.2 U/mg protein) and LP_30_/LR_30_ at intermediate levels (1.45 and 1.51 U/mg protein). These results indicate that LM_20_ and WO_20_ enhanced intestinal immune enzyme activities, while SP_10_ was the least effective.

Expression of inflammatory cytokines is presented in Figure 5C–E. TNF‐α and IL‐1β expression was significantly reduced in LM_20_ and WO_20_ (0.68 and 0.61 fold) compared with the control and SP_10_ (1.02 fold) (*p* < 0.05). LP_30_ and LR_30_ (0.85 and 0.82 fold) were intermediate. In contrast, IL‐10 expression was significantly higher in LM_20_ and WO_20_ (1.62 and 1.74 fold) relative to the control and SP_10_ (0.95 fold), with LP_30_ and LR_30_ showing moderate increases (1.28 and 1.35 fold). Overall, LM_20_ and WO_20_ promoted an anti‐inflammatory expression profile, while SP_10_ remained comparable to the control.

Tight junction gene expression is shown in Figure 5F–H. Claudin, occludin, and ZO‐1 were significantly upregulated in LM_20_ and WO_20_ (1.45–1.72 fold) compared with SP_10_ (0.98–1.07 fold) (*p* < 0.05). LP_30_ (1.20–1.30 fold) and LR_30_ (1.25–1.35 fold) showed moderate increases. These findings suggest that LM_20_ and WO_20_ improved intestinal barrier integrity, whereas LP_30_ and LR_30_ provided moderate benefits, and SP_10_ showed no improvement over the control.

In summary, diets containing 20% LM or 20% WO consistently enhanced intestinal immune enzyme activity, suppressed proinflammatory cytokine expression, increased anti‐inflammatory IL‐10, and upregulated barrier‐related genes. LP_30_ and LR_30_ produced moderate effects, while SP_10_ remained largely similar to the control.

### 3.6. Gut Microbiota Analysis

**Figure 6:**
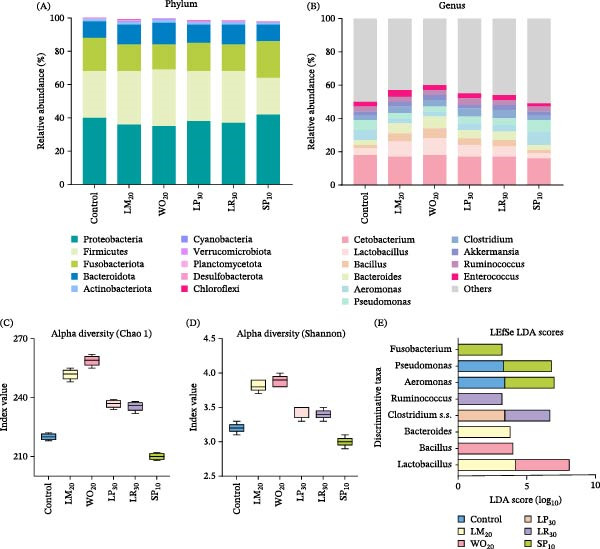
Gut microbiota analysis of koi carp fed experimental diets with selected duckweed inclusions. (A) Relative abundance of intestinal microbiota at the phylum level. (B) Relative abundance of intestinal microbiota at the genus level (top 10 genera shown; others grouped as “Others”). (C) Alpha‐diversity indices (Chao1 richness) of gut microbiota. (D) Alpha‐diversity indices (Shannon diversity) of gut microbiota. (E) Linear discriminant analysis effect size (LEfSe) identifying discriminative taxa among groups (LDA score > 3.0, log10). Values are presented as mean ± SD (*n* = 5). Different superscript letters indicate significant differences among groups (*p* < 0.05).

The intestinal microbiota composition of koi carp fed different duckweed‐based diets was further characterized at both the phylum and genus levels (Figure 6A, B). At the phylum level, Proteobacteria, Firmicutes, Fusobacteriota, and Bacteroidota dominated across all groups, together accounting for >85% of total sequences. Compared with the control, LM_20_ and WO_20_ increased the relative abundance of Firmicutes (32%–34%) while reducing Proteobacteria (35%–36%), whereas SP_10_ exhibited the opposite pattern with higher Proteobacteria (42%) and lower Firmicutes (22%). LP_30_ and LR_30_ showed intermediate distributions, remaining broadly similar to the control group. Minor phyla such as Actinobacteriota, Cyanobacteria, and Verrucomicrobiota were detected at low abundance (<2%).

At the genus level, distinct shifts were evident (Figure 6B). LM_20_ and WO_20_ enriched beneficial genera such as *Lactobacillus* (9%–10%), *Bacillus* (5%–6%), and *Bacteroides* (6%–7%) while reducing potential opportunistic pathogens including Aeromonas and Pseudomonas. In contrast, SP_10_ and the control group were characterized by higher proportions of *Aeromonas* (8%) and *Pseudomonas* (7%), together with reduced beneficial genera. LP_30_ and LR_30_ were enriched in *Clostridium sensu stricto* and *Ruminococcus*, reflecting intermediate effects.

Alpha‐diversity indices confirmed these compositional differences (Figure 6C, D). Both Chao1 richness and Shannon diversity were significantly higher in LM_20_ and WO_20_ (Chao1 248–255; Shannon 3.7–3.8) compared with the control (Chao1 218; Shannon 3.1) and SP_10_ (Chao1 208; Shannon 3.0) (*p* < 0.05). LP_30_ and LR_30_ maintained moderate diversity levels, falling between the high‐diversity (LM_20_ and WO_20_) and low‐diversity (SP_10_) groups.

LEfSe analysis further identified discriminatory taxa among treatments (Figure 6E). LM_20_ and WO_20_ were strongly associated with Lactobacillus, Bacillus, and Bacteroides (LDA score > 4.0), consistent with improved nutrient digestibility and immune function. SP_10_ and control diets were associated with Aeromonas, Pseudomonas, and Fusobacterium (LDA 3.2–3.6), aligning with their poorer growth and immune outcomes. LP_30_ and LR_30_ were discriminated by moderate enrichment of *C. sensu stricto* and *Ruminococcus* (LDA 3.2–3.4).

Taken together, these results demonstrate that 20% replacement of fishmeal with LM or WO promotes a more diverse and beneficial intestinal microbiota, whereas SP_10_ disrupts microbial balance by favoring opportunistic pathogens. LP_30_ and LR_30_ exerted intermediate effects, supporting moderate microbiota stability.

## 4. Discussions

This study demonstrated that partial replacement of fishmeal with duckweed had species‐specific effects on koi carp performance, nutrient utilization, pigmentation, and health status. The overall findings emphasize the dual role of duckweed as a sustainable protein source and a functional feed additive.

### 4.1. Nutritional Feasibility of Duckweed as a Fishmeal Replacer

It should be clarified that in the present study, duckweed was incorporated on a w/w basis rather than on a strict protein substitution basis; however, all diets were formulated to be isonitrogenous and isolipidic. Taken together with our growth and digestibility data, the balance of recent evidence indicates that moderate inclusion of duckweed (15%–20% of diet, corresponding to a proportional reduction of fishmeal on a weight basis) is nutritionally feasible in cyprinids and, in several cases, salmonids. In common carp, graded LM at 15%–20% improved WG, FCR, and digestive enzyme activity and enhanced muscle EAA and n‐3 LC‐PUFA accrual, in part via upregulation of elongase/desaturase genes, mechanistic support for the performance we observed at LM_20_ (and WO_20_) without an FCR penalty [30]. In rainbow trout, 20% protein substitution with LM had no adverse effects on growth or fillet proximate/fatty acid profiles, corroborating that well‐formulated duckweed diets can match conventional protein sources at moderate inclusion [31]. Across *Lemnaceae*, recent syntheses emphasize high‐quality amino acid profiles (notably lysine; methionine is typically adequate when diets are balanced) and good protein bioavailability when cultivation and processing are controlled, features aligning with the elevated body protein and EAA retention we recorded with LM_20_/WO_20_ [32, 33]. Finally, our finding that LP_30_ remained acceptable but below LM_20_ mirrors species/inclusion‐level contingencies reported in the literature, where performance tiers often follow species identity and dose. Collectively, these data support duckweed, particularly *Lemna* and *Wolffia*, at 20% as a nutritionally credible partial fishmeal replacer for koi carp.

Nutritional feasibility nonetheless depends on processing and specification control. Studies show that extrusion and related thermal/mechanical treatments improve starch/protein digestibility and reduce ANFs, explaining why moderate inclusions perform well, while higher doses (or minimally processed meals) may depress growth [34]. Species differences also matter: with SP, broken‐line regressions placed inflection points near 10%–13% for final weight/SGR in carp, consistent with our observation that SP_10_ was tolerable, whereas ≥30% impaired growth, underscoring the need to cap inclusion by species and to prefer *Lemna/Wolffia* where possible [35]. From a quality‐assurance perspective, duckweed protein concentrates have cleared EU safety assessments when produced under controlled conditions, although manganese carry‐over has been flagged for some WO ingredients, highlighting the importance of water/source control and compositional specs [36, 37]. In practice, formulating isonitrogenous/isoenergetic, amino‐acid–balanced diets, applying adequate processing, and restricting inclusion to ~20% for *Lemna*/*Wolffia* (≤10%–15% for *Spirodela*) provide a robust operating window in which duckweed reliably replaces a portion of fishmeal without compromising growth or feed efficiency.

It should also be noted that the nutritional value of duckweed is strongly affected by cultivation environment, including nutrient availability, light regime, water quality, and harvest stage. These factors can alter crude protein, amino acid balance, carotenoid accumulation, mineral composition, and ANF content and therefore may partly explain the species‐dependent responses observed in the present study. For this reason, the proximate composition and ANF contents of each duckweed meal were determined before diet formulation, and caution is warranted when extrapolating the present inclusion thresholds to duckweed biomass produced under different growth conditions.

We acknowledge that inclusion of an additional unsupplemented control diet could have provided further information on the independent contribution of crystalline amino acid supplementation. However, the objective of the present study was not to evaluate amino acid deficiency per se but to assess duckweed as a practical fishmeal‐reducing ingredient under nutritionally balanced diet conditions. For this reason, amino acid balancing was applied consistently across diets to improve comparability among treatments and to better reflect practical aquafeed formulation.

### 4.2. Functional Roles of Duckweed Beyond Nutrition

Beyond supplying digestible protein, the LM_20_ and WO_20_ diets acted as functional ingredients that improved pigmentation and redox–immune homeostasis in koi. The higher *a* 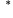 and *b* 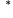 values paralleled greater deposition of xanthophylls (zeaxanthin and lutein) and astaxanthin, consistent with the unusually zeaxanthin‐rich carotenoid profile of *Lemnaceae* and with strategies that deliberately raise zeaxanthin in duckweed biomass before harvest [32, 38]. In fish, dietary carotenoids are not only chromophores but also modulators of oxidative physiology; they upregulate antioxidant defenses and can improve stress resilience when provided at appropriate doses [39–42]. Our concurrent increases in SOD and CAT with lower MDA in LM_20_/WO_20_ align with these functions and with reports that *Wolffia*/*Lemna* contain tocopherols and xanthophylls that contribute to strong in vitro and in vivo antioxidant capacity [43–45]. From a formulation perspective, these findings suggest that duckweed can replace synthetic pigments in ornamental feeds while delivering antioxidant micronutrients that support coloration quality and oxidative balance.

LM_20_ and WO_20_ also yielded a more favorable intestinal immune–barrier phenotype (higher lysozyme/PO, reduced TNF‐α/IL‐1β, increased IL‐10, and upregulated claudin/occludin/ZO‐1). Such profiles are compatible with the polyphenol‐rich, flavonoid‐rich nature of duckweeds—dominated by C‐glycosyl flavones (e.g., luteolin/apigenin derivatives) with documented antioxidant and immunomodulatory activities [45–47]. Recent synthesis articles in aquaculture further highlight that natural antioxidants (carotenoids, polyphenols, and vitamins) can attenuate mucosal inflammation and reinforce epithelial integrity, offering a mechanistic basis for the gene‐expression patterns observed here [34, 48–50]. Taken together, our results indicate that species‐selected duckweed (LM and WO) functions as a dual‐purpose ingredient, providing protein while delivering bioactives that enhance pigmentation and promote anti‐inflammatory, barrier‐supportive intestinal conditions, thereby addressing both esthetic and welfare targets central to ornamental aquaculture.

### 4.3. Implications for Sustainable Aquaculture and Ornamental Fish Production

From a systems perspective, adopting LM_20_/WO_20_ does more than displace a portion of fishmeal. Although the duckweed biomass used in the present study was produced under controlled greenhouse tank conditions rather than on aquaculture effluents, duckweed remains attractive for future circular feed strategies because of its rapid biomass production and high nitrogen–phosphorus uptake capacity. When cultivated on nutrient‐rich waste streams, duckweed may help recover dissolved nutrients while generating biomass for feed applications. Recent pilot and semicommercial studies show duckweed units recovering substantial N and P in IMTA configurations, thereby valorizing wastewater and reducing the environmental footprint of fish production (e.g., duckweed–bivalve–fish couplings) [51]. Likewise, global assessments of nature‐based water treatment highlight *Lemnaceae* as among the most efficient macrophytes for polishing aquaculture and municipal effluents, supporting water reuse and on‐site biomass generation [52]. At the feed‐mill level, current reviews converge that duckweed (together with microalgae) is among the few scalable, nutrient‐dense aquatic crops capable of reducing pressure on marine ingredients while maintaining feed functionality, provided that cultivation and processing are standardized for amino acid consistency and digestibility [8, 15]. Emerging RAS studies also suggest that duckweed modules integrated with other filter feeders can improve water quality and yield a usable feed fraction, further tightening the nutrient loop [53]. In practical terms, our LM/WO inclusion at ~20% aligns with these systems‐level insights: It reduces fishmeal dependence, improves pigment and health endpoints valued in ornamental markets, and is compatible with on‐site biomass supply chains that convert waste nutrients into value.

For ornamental aquaculture specifically, duckweed’s bioactive profile (xanthophylls, polyphenols, and vitamins) provides a credible natural‐additive strategy to replace or reduce synthetic pigments and antioxidant premixes while supporting coloration, oxidative balance, and mucosal integrity, attributes with direct market and welfare implications. Adoption, however, should proceed with quality assurance and regulatory awareness. In the EU, fresh Wolffia (*W. arrhiza*/WO) has been authorized as a novel food, facilitating its positioning as a safe edible crop, whereas WO powder raised safety questions primarily related to manganese exposure, underscoring the need to control culture media, harvest timing, and specifications [37]. These guardrails are directly relevant to ornamental feed manufacturing: Standardized cultivation and processing (e.g., water source management and dewatering/fermentation where appropriate) help stabilize carotenoid and protein yields, minimize heavy‐metal carryover, and ensure lot‐to‐lot consistency. In sum, positioning LM/WO at 20% as a dual‐purpose, site‐grown ingredient, protein with functional bioactives, offers a practicable route to greener ornamental feeds that meet performance targets while advancing circular‐economy objectives; scaling should focus on process control, specification setting, and supply‐chain integration rather than ingredient substitution alone.

### 4.4. Gut Microbiota Modulation by Duckweed Inclusion

Our gut microbiota analysis revealed that dietary inclusion of Lemna and Wolffia at 20% (LM_20_ and WO_20_) provoked marked shifts in intestinal microbial communities, which likely contribute mechanistically to the improved nutrient utilization, immune response, and growth observed in these groups. In particular, LM_20_ and WO_20_ increased the relative abundance of phyla such as Firmicutes and Actinobacteriota while reducing dominance of Proteobacteria and Fusobacteriota, echoing trends seen in plant‐polyphenol studies in koi carp, where modulation of gut phylum composition toward greater Firmicutes:Proteobacteria ratios correlated with reduced inflammatory cytokine expression [54, 55]. At the genus level, enrichment of *Lactobacillus*, *Bacillus*, and *Bacteroides* under LM_20_/WO_20_ is consistent with their known roles in fermentative metabolism, short‐chain fatty acid (SCFA) production, and immunomodulation in fish [56–58]. Conversely, diets such as SP_10_ and the control showed higher relative abundances of *Aeromonas* and *Pseudomonas*, which are often regarded as opportunistic or conditionally pathogenic taxa, especially under less optimal diet formulations [59].

Alpha diversity (Chao1 and Shannon) was significantly elevated in LM_20_/WO_20_ compared to control and SP_10_, indicating not just compositional shifts but also greater richness and evenness; such increases in diversity are frequently associated with resilience to gut disturbances and enhanced metabolic flexibility [60]. LEfSe biomarkers identified *Lactobacillus* and *Bacillus* as discriminant taxa for LM_20_/WO_20_, supporting the functional microbial signature that underpins improved digestion, barrier integrity, and immune balance. Taken together, the duckweed‐driven microbiota remodeling in koi carp, especially under diets with Lemna or Wolffia at ~20%, suggests a dual mechanism: direct nutritional contributions plus microbial‐mediated benefits for gut health and host performance.

## 5. Conclusion

This study demonstrates that partial replacement of fishmeal with duckweed, particularly LM and WO at 20%, is both nutritionally feasible and functionally beneficial for koi carp. These inclusions sustained growth and feed efficiency, enhanced protein digestibility and amino acid retention, improved pigmentation through higher carotenoid deposition, and strengthened antioxidant and immune responses. Notably, LM_20_ and WO_20_ diets promoted favorable intestinal immune–barrier profiles and reshaped the gut microbiota toward greater diversity and enrichment of beneficial taxa such as *Lactobacillus* and *Bacillus* while reducing opportunistic pathogens. Collectively, these outcomes highlight duckweed as a dual‐purpose feed ingredient, simultaneously providing sustainable protein and functional bioactives. For ornamental aquaculture, such effects translate into improved coloration, resilience, and welfare, while integration of duckweed cultivation with nutrient recycling supports circular, environmentally sustainable production systems.

## Author Contributions

Yingjie Song conceived and supervised the study and wrote the manuscript. Zhangli Hu, Shasha Luo, Xinyan Yang, Ruimin He, Xuewei Yang, and Yinglin Lu revised the manuscript. Shuang Li wrote and revised the manuscript.

## Funding

This work was financially supported by the Guangdong Foundation for Program of Science and Technology Research (Grant 2020B1111530002) and the Special Funds Program of Guangdong Academy of Sciences (Grant 3022180002).

## Disclosure

All the authors read and approved the final manuscript.

## Conflicts of Interest

The authors declare no conflicts of interest.

## Supporting Information

Additional supporting information can be found online in the Supporting Information section.

## Supporting information


**Supporting Information** The supporting information file includes additional Figure S1 and Tables S1–S4 that support the main findings of this study. These materials provide extended experimental data, statistical analyses, and supplementary validation that could not be fully presented in the main text due to space limitations.

## Data Availability

The data that support the findings of this study are availabe from the corresponding author upon reasonable request.
