# Supplementary material for: Duckweed (Lemnaceae) as a Functional Protein Ingredient in Koi Carp Diets: Species‐Dependent Effects on Growth, Pigmentation, Antioxidant Status, and Gut Health
Source: Aquac Nutr. 2026 Jun 7;2026:3873558. doi: 10.1155/anu/3873558 (PMC13243698; doi:10.1155/anu/3873558)
Supplement: Supplementary file 1 — Supporting Information The supporting information file includes additional Figure S1 and Tables S1–S4 that support the main findings of this study. These materials provide extended experimental data, statistical analyses, and supplementary validation that could not be fully presented in the main text due to space limitations. [file ANU-2026-3873558-s001.docx]

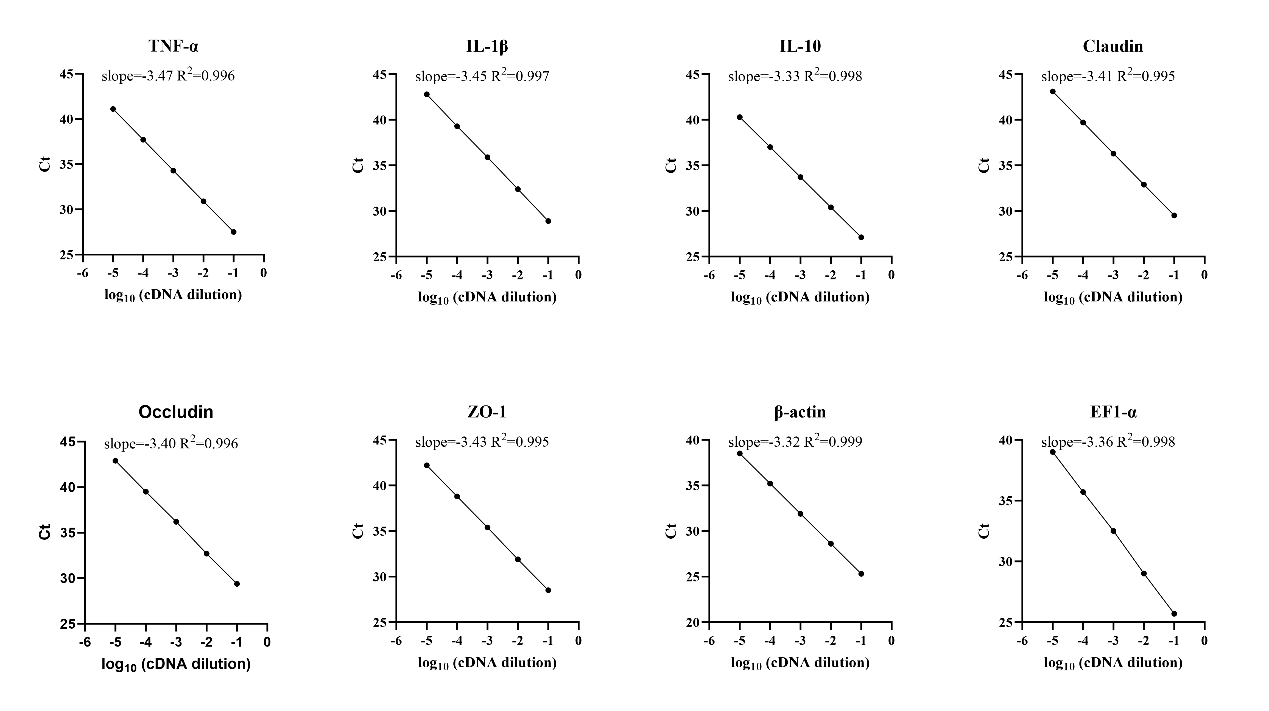


**Figure S1** qPCR standard curves for target and reference genes. E = (10^(–1/slope) – 1) × 100 %.

**Table S1.** Composition and proximate analysis of the experimental diets used in Phase I (five duckweed species × five replacement levels).

| Ingredient (g/kg dry diet) | Fishmeal | Soybean meal | Duckweed meal | Wheat flour | Corn gluten meal | Fish oil | Soybean oil | Vitamin-mineral premix | Lysin | Methionine | Total (g) |
| --- | --- | --- | --- | --- | --- | --- | --- | --- | --- | --- | --- |
| 0% (control) | 400 | 250 | 0 | 200 | 80 | 25 | 20 | 20 | 2.5 | 2.5 | 1000 |
| 10% replacement | 360 | 250 | 40 | 200 | 80 | 25 | 20 | 20 | 2.5 | 2.5 | 1000 |
| 20% replacement | 320 | 250 | 80 | 200 | 80 | 25 | 20 | 20 | 2.5 | 2.5 | 1000 |
| 30% replacement | 280 | 250 | 120 | 200 | 80 | 25 | 20 | 20 | 2.5 | 2.5 | 1000 |
| 40% replacement | 240 | 250 | 160 | 200 | 80 | 25 | 20 | 20 | 2.5 | 2.5 | 1000 |

Note: Vitamin–mineral premix supplied per kg diet: vitamin A 5000 IU, vitamin D₃ 1000 IU, vitamin E 50 mg, vitamin K₃ 10 mg, vitamin C 200 mg, etc.

Proximate composition (mean ± SD): crude protein ≈ 40 ± 0.3%, crude lipid ≈ 6 ± 0.2%, ash ≈ 8 ± 0.3%, gross energy ≈ 18.5 MJ kg⁻¹.

**Table S2.** Ingredient composition (g/kg dry diet) and proximate analysis of Phase II diets.

| **Ingredient (g/kg)** | **Cont.0** | **LM_20_** | **WG_20_** | **LP_30_** | **SP_10_** | **LRP_30_** |
| --- | --- | --- | --- | --- | --- | --- |
| Fishmeal | 400 | 320 | 320 | 280 | 360 | 280 |
| Soybean meal | 250 | 250 | 250 | 250 | 250 | 250 |
| Duckweed meal (LM) | 0 | **80** | 0 | 0 | 0 | 0 |
| Duckweed meal (WG) | 0 | 0 | **80** | 0 | 0 | 0 |
| Duckweed meal (LP) | 0 | 0 | 0 | **120** | 0 | 0 |
| Duckweed meal (SP) | 0 | 0 | 0 | 0 | **40** | 0 |
| Duckweed meal (LRP) | 0 | 0 | 0 | 0 | 0 | **120** |
| Wheat flour | 200 | 200 | 200 | 200 | 200 | 200 |
| Corn gluten meal | 80 | 80 | 80 | 80 | 80 | 80 |
| Fish oil | 25 | 25 | 25 | 25 | 25 | 25 |
| Soybean oil | 20 | 20 | 20 | 20 | 20 | 20 |
| Vitamin–mineral premix | 20 | 20 | 20 | 20 | 20 | 20 |
| L-lysine (crystalline) | 2.5 | 2.5 | 2.5 | 2.5 | 2.5 | 2.5 |
| DL-methionine (crystalline) | 2.5 | 2.5 | 2.5 | 2.5 | 2.5 | 2.5 |
| Total (g) | 1000 | 1000 | 1000 | 1000 | 1000 | 1000 |

Note: Proximate composition of finished feeds (mean ± SD): crude protein 40.0 ± 0.3%, crude lipid 6.1 ± 0.2%, ash 8.0 ± 0.3%, gross energy 18.5 ± 0.3 MJ kg⁻¹.
Vitamin–mineral premix per kg diet: vitamin A 5,000 IU; vitamin D₃ 1,000 IU; vitamin E 50 mg; vitamin K₃ 10 mg; vitamin C 200 mg; B-vitamins and trace minerals at standard levels.

**Table S3.** Proximate and amino-acid composition of five duckweed species used in diet formulation (dry-matter basis).

| **Duckweed species** | **Crude protein (%)** | **Crude lipid (%)** | **Ash (%)** | **Crude fiber (%)** | **NFE (%)** | **Lys (g 100 g⁻¹ CP)** | **Met (g 100 g⁻¹ CP)** |
| --- | --- | --- | --- | --- | --- | --- | --- |
| *L. minor* | 38.2 ± 0.6 | 4.5 ± 0.3 | 10.2 ± 0.2 | 8.4 ± 0.5 | 38.7 ± 0.4 | 6.8 | 2.2 |
| *W. globosa* | 41.0 ± 0.8 | 5.0 ± 0.4 | 9.5 ± 0.3 | 7.8 ± 0.4 | 36.7 ± 0.5 | 7.1 | 2.3 |
| *L. punctata* | 36.5 ± 0.7 | 3.9 ± 0.2 | 11.0 ± 0.3 | 9.5 ± 0.6 | 39.1 ± 0.5 | 6.4 | 2.1 |
| *S. polyrhiza* | 34.8 ± 0.9 | 3.5 ± 0.3 | 12.3 ± 0.4 | 10.1 ± 0.6 | 39.3 ± 0.6 | 6.2 | 2.0 |
| Rootless  *L. punctata* | 37.2 ± 0.5 | 4.1 ± 0.2 | 10.8 ± 0.3 | 9.0 ± 0.5 | 38.9 ± 0.4 | 6.6 | 2.1 |

**Table S4.** Primer sequences, amplicon length, and efficiency (%)

| **Gene** | **Primer Sequence (5′–3′)** | **Amplicon (bp)** | **E (%)** | **R²** |
| --- | --- | --- | --- | --- |
| **TNF-α** | F: TGCCAGTGTGTGAAAGGAGT R: CTCTGCCATGTTCTGAGTGC | 154 | 97.9 | 0.997 |
| **IL-1β** | F: CAGTCTCTCCTGTCCTGGTG R: TGGTGATGGTGTTCTTGGTG | 182 | 95.0 | 0.996 |
| **IL-10** | F: GCTGACGATGACAGAGCTTC R: CGTCTTGATGTTGTTGGTGG | 171 | 99.7 | 0.998 |
| **Claudin** | F: TTGGTGGTGCTGTTCTTTGC R: ATGGTCTTGCTGCTGTTGTC | 160 | 96.5 | 0.995 |
| **Occludin** | F: CGACATCGTGGAGTTCAAGA R: GGTTGGTTCTGTGCTGTTGT | 143 | 97.0 | 0.996 |
| **ZO-1** | F: AAGTGGCTGATGAGGATGGA R: CTTGGTCTTGGTGCTGTGTT | 175 | 95.8 | 0.995 |
| **β-actin (ref 1)** | F: TCCACCTTCCAGCAGATGTG R: AGGTCCTTACGGATGTCCAC | 152 | 100.1 | 0.999 |
| **EF1-α (ref 2)** | F: TGACATCGTTGGTGGTGACA R: CCTTGTAGACGACCTTCTGG | 167 | 98.5 | 0.998 |
